# Sparse reduced-rank regression for integrating omics data

**DOI:** 10.1186/s12859-020-03606-2

**Published:** 2020-07-03

**Authors:** Haileab Hilafu, Sandra E. Safo, Lillian Haine

**Affiliations:** 1 Department of Business Analytics and Statistics, University of Tennessee, Knoxville, 37996 TN USA; 2 Division of Biostatistics, University of Minnesota, Minneapolis, 55455 MN USA

**Keywords:** Integrative analysis, Multi-view data, Reduced rank regression, High dimensional data

## Abstract

**Background:**

The problem of assessing associations between multiple omics data including genomics and metabolomics data to identify biomarkers potentially predictive of complex diseases has garnered considerable research interest nowadays. A popular epidemiology approach is to consider an association of each of the predictors with each of the response using a univariate linear regression model, and to select predictors that meet a priori specified significance level. Although this approach is simple and intuitive, it tends to require larger sample size which is costly. It also assumes variables for each data type are independent, and thus ignores correlations that exist between variables both within each data type and across the data types.

**Results:**

We consider a multivariate linear regression model that relates multiple predictors with multiple responses, and to identify multiple relevant predictors that are simultaneously associated with the responses. We assume the coefficient matrix of the responses on the predictors is both row-sparse and of low-rank, and propose a group Dantzig type formulation to estimate the coefficient matrix.

**Conclusion:**

Extensive simulations demonstrate the competitive performance of our proposed method when compared to existing methods in terms of estimation, prediction, and variable selection. We use the proposed method to integrate genomics and metabolomics data to identify genetic variants that are potentially predictive of atherosclerosis cardiovascular disease (ASCVD) beyond well-established risk factors. Our analysis shows some genetic variants that increase prediction of ASCVD beyond some well-established factors of ASCVD, and also suggest a potential utility of the identified genetic variants in explaining possible association between certain metabolites and ASCVD.

## Background

Advances in technologies and data collection processes have resulted in multiple high dimensional data types being measured on the same subjects. For instance, in biomedical research, these data types include genomics, metabolomics, proteomics, and transcriptomics. While each of these data types provide a different snapshot of the underlying biological system, it is being increasingly recognized that combining these data types can reveal complex relationships that may not be unraveled from individual analyses. For instance, the integration of genomic and metabolomic/proteomic data can provide valuable insight into key genomic loci that influence human plasma levels associated with complex diseases [1]. This is of great interest because genomic studies including genome wide association studies (GWAS) have revealed that the majority of disease-causing single nucleotide polymorphisms (SNPs) lie in noncoding regions of the genome [2], making it difficult to know their functional implications. While individual genomic variants identified through GWAS can be tested experimentally, this approach is complicated by the modest effects of the identified variants and the fact that we may not know the specific gene driving the genomic association [3]. Integration of genomics data with other omics data can therefore enable us to identify genomic variants that could generate hypotheses for the genomic architecture of the underlying disease, or could identify variants that have the potential to improve clinical factors. Since the metabolome is considered as the end product of all genetic, epigenetic, and environment activities [4, 5], linking metabolite levels in human blood samples with genomics data can help shed light on complex disease-causing genomic variants. Additionally, tying genomic variants to metabolite levels can identify metabolites that can be used as biomarkers or potential targets for drug discovery [1]. A review of studies that combine genomics and metabolomics data can be found in [3]. In a recent study [1], genomics data were linked with protein levels known to be associated with cardiovascular disease (CVD) and many new gene locus-protein associations were unraveled, providing new insight into CVD risk pathophysiology [1].

However, integrating genomics and metabolomics data, for instance, to identify important disease-associated biomarkers is complicated by the high-dimensional nature of each omics data. The popular epidemiological approach to relate genomics with metabolomics or proteomics data is to consider an association of each of the genetic variant with each of the metabolites using a univariate linear regression model, and to select genetic variants that meet a priori specified significance level [1, 6, 7]. Specifically, the following linear regression model is considered:

1 $$ \mathbf{y}_{j} = \mathbf{x}_{i} {c}_{i} + \mathbf{e}_{j}  $$

where **y**_*j*_,*j*=1,…,*q* is a *n*×1 vector of metabolic features or protein expression levels for *n* subjects, **x**_*i*_,*i*=1,…,*p* is a *n*×1 vector of SNPs for *n* subjects, c _*i*_ is the unknown coefficient for the *i*th SNP, e _*j*_ is a vector of random noise, *q* denotes the number of responses (metabolic features or protein levels), and *p* denotes the number of predictors (SNPs). The above approach is limiting because larger sample size is usually required to identify associated biomarkers, which is costly. Furthermore, it assumes variables for each data type are independent, and thus ignores correlations that exist between variables both within each data type and across the data types. Additionally, genomic studies show that most genetic variants have modest effect on complex diseases, suggesting the need for methods that model multiple SNPs simultaneously in association studies. These limitations lead us to consider the following multivariate linear regression model

2 $$ \mathbf{Y} = \mathbf{X} \mathbf{C} + \mathbf{E},  $$

where **Y** is *n*×*q* matrix containing all the responses (e.g., all metabolites), **X** is *n*×*p* matrix of predictors (e.g., SNPs), **C** is a *p*×*q* matrix of unknown coefficients, and **E** is *n*×*q* matrix of random noise. Our goal is then to estimate the matrix of unknowns **C**, and to identify multiple relevant predictors that are simultaneously associated with the responses, and which potentially could predict complex diseases. From a statistical point of view, the discovery of biomarkers is best cast as a variable selection problem, where “variable” refers to the genetic loci or metabolites. Variable selection in omics data is complicated by the high-dimensional nature of each of the omics data.

When we use (2) to model genomics and metabolomics/proteomics data, with a large number of responses and predictors, the number of unknown parameters that need to be estimated in **C**, i.e. *pq*, can quickly exceed the sample size *n*. To overcome this problem, researchers have considered two important types of structural assumptions that induce lower-intrinsic-dimension on **C**. The first is *low-rankness* where the rank of **C** is assumed to be much smaller than its matrix dimension of *min*(*p,q*). That is, it is assumed that *rank*(**C**)=*r*<*mifvn*(*p,q*). Then, counting the parameters in the singular value decomposition of **C**, we observe that only *r*(*p*+*q*−*r*) free parameters need to be estimated, which can be substantially lower than *pq* for low values of *r*. This structure is referred to as the *reduced-rank regression* (RRR) and has been widely used in variety of applications [8, 9]. Reduced-rank estimation is often obtained by introducing penalties that are proportional to the eigenvalues of the coefficient matrix or its rank, see, for example, [10–15].

The second structural assumption is the so called *sparsity* where only a small subset, *s*, out of the *p* predictors are assumed to contribute to the variation of the responses. Removing the *i*th predictor from model (2) is equivalent to setting the *i*th row in **C** to zero. Vectorizing both sides of model (2) yields a univariate response regression model. Thus, one can view the rows of **C** as groups of coefficients in the transformed model and set them to zero by any group selection method developed for univariate response regression models. Thus, the effective number of parameters is *sq*, which is smaller than the unrestricted *pq*, but may be higher than *r*(*p*+*q*−*r*), especially if the rank of **C** is low. Proposals that use penalties that induce *row-sparsity* include, among others, [16–21]. For either structure, researchers have sought to understand how a given statistical estimation depends on the model parameters and on how to achieve optimal estimation without the knowledge of the rank *r* or the sparsity level *s*. In this article, we propose a new method that induces both *row-sparsity* and *low-rankness*, and leads to meaningful dimension reduction and variable selection.

### Motivating data: an atherosclerosis disease study

We motivate our work using data from the Emory/Georgia Tech Predictive Health Institute (PHI) study. The PHI is longitudinal study of healthy employees from Emory University and Georgia Tech that began in 2005 with the aim of collecting health factors that could be used to optimize and maintain health rather than treating disease. With this in mind, we seek to identify genomic risk factors that are correlated with motabolite and which could be used for predicting 10-year risk of ASCVD. ASCVD is a chronic inflammatory disease as well as a disorder of lipid metabolism [22]. It is a complex disease of many risk factors including genetic risk factors. Many genetic studies have been conducted to identify genetic variants and genes that many be implicated in ASCVD [23]. However, the functional implications of these SNPs and genes are not well-understood. Linking metabolomic data with genomic data can help shed light on genetic loci influencing ASCVD. Additionally, tying genetic loci to metabolomics can identify metabolites that can be used as biomarkers for ASCVD [1]. In light of the above, we seek to use metabolites to guide selection of SNPs that may be associated with ASCVD, and to also explore the potential utility of these genetic variants in explaining possible association between certain metabolites, and ASCVD risk.

### Main contributions

This paper makes two main contributions. First, we propose a new computationally efficient convex formulation to estimate the coefficient matrix in (2) that takes advantage of the potential presence of *low-rankness* and *sparsity*. The proposed convex formulation is computationally efficient, and can be solved using readily available solvers. It is also shown to yield competitive numerical performances (in estimation, prediction, and variable selection) under a variety of model parameter settings when compared with state-of-the-art methods in the literature. Specifically, we observe that the superior results of the proposed method, in estimation and variable selection, are more pronounced when the number of responses and predictors are much higher than the sample size. This is encouraging to us since our motivating problem, and many integrative genomics analysis problems, fall under this regime. Second, atherosclerosis cardiovascular disease is a major health-economic burden in USA, and beyond, and the problem of identifying other non-traditional risk factors beyond well-established factors remains an important scientific problem and active research area. We aim to contribute to the body of knowledge in this field through the use of innovative statistical methods such as the ones proposed here. We therefore present careful analyses of data from healthy adults with low- vs moderate- to high-risk for developing atherosclerosis cardiovascular disease in the future using genomics, metabolomics, clinical, and demographic data, permitting us to identify genetic variants that increase atherosclerosis cardiovascular disease risk beyond established risk factors. Additionally, we explore the potential use of these genetic variants in explaining possible association of certain metabolites with atherosclerosis cardiovascular disease.

## Method

### Reduced-rank regression

Let $\left \{ \mathbf {x}_{i}^{\top },\mathbf {y}_{i}^{\top } \right \}_{i=1}^{n}$ denote an available *n* i.i.d. samples. In the sequel, we denote the predictor and response data matrices by **X** and **Y**, respectively. Suppose that **C** is of lower rank, *r* = rank(**C**)< min(*p,q*), and that we have a *q*×*r* orthonormal matrix **A** whose columns span the right singular subspace of **C**. That is, we have a *q*×*r* orthonormal matrix **A** such that for some *p*×*r* matrix **B**,**C**=**BA**^⊤^. Then, post-multiplying both sides by **A**, we can re-write model (2) as

3 $$ \mathbf{Y} \mathbf{A} = \mathbf{X} \mathbf{B} + \mathbf{E}\mathbf{A},  $$

where **XB** is of reduced dimension with only *r* components that can be interpreted as unobservable latent factors that drive the variation in the responses. This re-parametrization also indicates that **A** spans the right singular subspace of **Y**. Therefore, if we had such a matrix **A**, we would fit a lower dimensional regression of **YA** on **X** to obtain an estimate of **B**, and use it to obtain an estimate for **C**. In the literature, model (3) is referred to as the *reduced-rank regression* model. Since the responses are modeled by *r*(*r*<*q*) common latent factors, we achieve dimensionality reduction of the predictors and expect that this modeling exercise takes the correlations among the *q* responses into account. There are a number of approaches of obtaining such a matrix **A**. For example, [17] and [24] consider the SVD, **C**=**UDV**^⊤^, where **U** and **V** are *p*×*r* and *q*×*r* matrices with orthonormal columns, respectively, and **D** is a *r*×*r* nonnegative diagonal matrix. [18] set **A** to be the *r*-dimensional right singular subspace of **Y**. We exploit both these approaches in this paper. On the other hand, [20], propose to seek such an **A** that leads to the best approximation of the signal matrix **XC**. That is, they seek a *q*×*r* orthogonal matrix **A** such that **XBA**^⊤^ is the best rank *r* approximation of the signal matrix **XC**.

The latent factors **XB** (*r* components) are lower dimensional than the original predictors. However, they are linear combinations of all the *p* original predictors. Therefore, while model (3) achieves dimension reduction of the predictors, it does not lead to variable selection. Recall that a predictor is unimportant in predicting the responses via model (2) if the corresponding row in **C** is zero. Thus, to achieve variable selection, row-sparse estimate of **C** is desirable. The following two key facts facilitate this using model (3): (*i*) if **C** has at most *s* non-zero rows, so does **B**=**CA**; (*ii*) the non-zero rows in **B** and **C** are the same. Thus, row-sparse estimate of **C** can be obtained by seeking row-sparse estimate of **B**.

### Our approach to sparse reduced-rank regression

Suppose that we have a matrix $\tilde {\mathbf {A}}$ as described above (we discuss how to obtain such a matrix in the implementation section below). We use the following optimization problem for a row-sparse estimation in reduced-rank regression (3):

4 $$ \widehat{\mathbf{B}} = \min_{\mathbf{B}} \sum_{j=1}^{p} \left\|\mathbf{b}_{j}\right\|_{2} \qquad \quad \text{ subject to } \qquad \quad \max_{1\leq j \leq p} \left \| \mathbf{X}^{\top}_{j} \left (\mathbf{Y} \tilde{\mathbf{A}} - \mathbf{X} \mathbf{B} \right) \right \|_{1} \leq \tau,  $$

where *τ*>0 is a tuning parameter that controls the sparsity level in $\widehat {\mathbf {B}}$. Large values of *τ* yield less sparse estimates and smaller values of *τ* yield more sparse estimates. The formulation in (4) can be thought of as a generalization of the dantzig selector [25] to a multivariate reduced-rank regression setting, with $\mathbf {Y}\tilde {\mathbf {A}}$ as the response and **X** as the predictor. This formulation, which yields row-sparse estimates, is desirable for the following reasons: (*i*) the solution to the optimization problem is unique up to a *r*×*r* orthogonal matrix; (*ii*) the set of important predictors obtained by solving the optimization problem is uniquely determined, where the important predictors are those that correspond to nonzero rows of the solution. Consequently, the solutions to (4) are determined up to an orthogonal transformation. Nonetheless, different solutions correspond to selection of the same set of predictors, hence the name *coordinate-independent* sparse reduced-rank regression (CISRRR).

### Implementation

We focus on the reduced-rank version of our proposal via (4), as this is the more useful method for the high-dimensional setting. The proposed method can be viewed as a two stage estimation approach. In the first stage, we seek an estimate of the right singular subspace of **C**, or the right singular subspace of **Y**, say $\widehat {\mathbf {A}}$. In the second stage, we solve (4) to obtain row-sparse estimate $\widehat {\mathbf {B}}$ of **B**. The corresponding row-sparse estimate of **C** is then obtained as: $\widehat {\mathbf {C}} = \widehat {\mathbf {B}}\widehat {\mathbf {A}}^{\top }$.


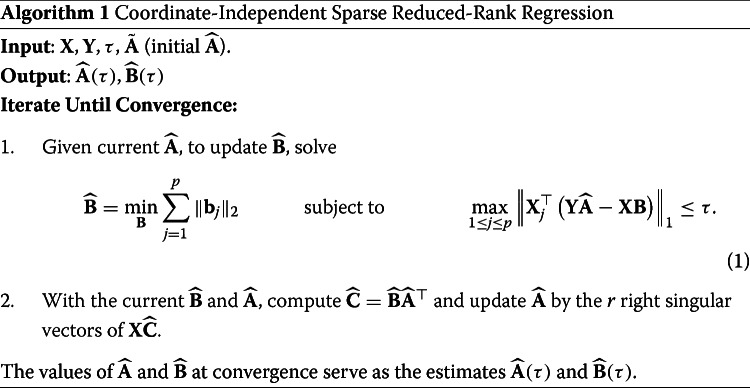


To understand the motivation behind the updating in step 2, note that if we knew **C**, we can think of the right singular subspace of **XC** as estimating the right singular subspace of **Y**. Therefore, this step can be thought of as encouraging the updating **A** and **B** such that **XC** approximates **Y**. Next, we discuss two approaches to obtain the initial estimate $\tilde {\mathbf {A}}$.

1. We can use the sample estimate for the right singular subspace of **Y** as the initial estimate of **A**. Let **Y**=**UDV**^⊤^, where $\mathbf {U}\in \mathbb {R}^{n \times r}, \mathbf {D}$ is a diagonal *r*×*r* matrix, and **V** is *q*×*r*. Set $\tilde {\mathbf {A}} = \mathbf {V}$ as the initial estimate.
2. Alternatively, we can use some regularization to obtain the initial estimate. Suppose that $\widehat {\mathbf {C}}^{\text {OLS}}$ is the nonsparse OLS estimate of **C**. That is, $\widehat {\mathbf {C}}^{\text {OLS}} = \left (\mathbf {X}^{\top }\mathbf {X}\right)^{-1}\mathbf {X}^{\top }\mathbf {Y}$. Perform a singular-value decomposition (SVD): $ \widehat {\mathbf {C}}^{\text {OLS}} = \mathbf {U}\mathbf {D}\mathbf {V}^{\top }$. Then, use the first *r* columns of **V** as the estimate $\tilde {\mathbf {A}}$. In the event that *n*<*p*, we use the ridge-type estimator instead of the OLS estimator, i.e. $\widehat {\mathbf {C}}^{\text {Ridge}} = \left (\mathbf {X}^{\top }\mathbf {X} + \mathbf {I}*\sqrt {\log p/n} \right)^{-1}\mathbf {X}^{\top }\mathbf {Y}$. For computational expediency, we also avoid having to invert the *p*×*p* matrix $\left (\mathbf {X}^{\top }\mathbf {X} + \mathbf {I}*\sqrt {\log p/n} \right)$. Instead, we use the tricks given in [26] and invert an *n*×*n* matrix. More specifically, let **X**=**UDV**^⊤^ represent the SVD of **X**; that is, **V** is *p*×*n* with orthogonal columns, **U** is *n*×*n* with orthogonal columns, and **D** a diagonal matrix with elements *d*_1_≥*d*_2_≥⋯≥*d*_*n*_≥0. [26] show that a computationally efficient estimate of the ridge estimator can be obtained as: $\widehat {\mathbf {C}}^{\text {Ridge}} = \mathbf {V} \left (\mathbf {R}^{\top }\mathbf {R} + \mathbf {I}*\sqrt {\log p/n} \right)^{-1}\mathbf {R}^{\top }\mathbf {Y}$, where the matrix **R** = **UD** is *n*×*n*, leading to a much less computationally expensive estimate.

All our empirical results are based on the first approach. However, we have conducted simulation studies to assess the performances using the second approach as well. The simulations showed that the two approaches yield comparable results. Once an initial $\tilde {\mathbf {A}}$ is specified, to solve the optimization problem in (??), we used CVX, a MATLAB package for specifying and solving convex optimization problems [27, 28]. Matlab codes that implement this algorithm are provided as online supplementary material (see Additional file 2).

### Tuning parameter selection

The tuning parameter *τ* in (??) controls the level of sparsity in $\widehat {\mathbf {B}}$, and hence in $\widehat {\mathbf {C}}$, and needs to be selected adaptively from the data. Notice that when $\tau >\max _{1\leq j \leq p} \left \| \mathbf {X}^{\top }_{j} \mathbf {Y} \widehat {\mathbf {A}} \right \|_{1} $, the optimization problem (??) yields a trivial solution, giving us an upper bound for *τ*. Therefore, we choose the optimal *τ* from the range $\left (0,\max _{1\leq j \leq p} \left \| \mathbf {X}^{\top }_{j} \mathbf {Y} \widehat {\mathbf {A}} \right \|_{1} \right)$ using *K*-fold cross validation. Specifically, for a given *τ*, we randomly split the available data {(**Y**,**X**)} into *K* roughly equal-sized non-overlapping groups of observations, which we denote by {(**Y**,**X**)}^*k*^,*k*=1,…,*K*. Let {(**Y**,**X**)}^−*k*^ be the data matrix leaving out {(**Y**,**X**)}^*k*^. With a given *τ*, we apply the proposed method on {(**Y**,**X**)}^−*k*^ to obtain an estimate of the coefficient matrix $\widehat {\mathbf {C}}^{k}(\tau)$. Then, we compute the *K*-fold mean squared prediction error (MSPE) as follows:

5 $$ \text{MSPE}(\tau) = \frac{1}{K}\sum_{k=1}^{K}\frac{\left \|\mathbf{X}^{k}\widehat{\mathbf{C}}^{k}(\tau) - \mathbf{Y}^{k} \right \|_{\text{F}}^{2}}{n_{k}q}  $$

where *n*_*k*_ is the number of observations in {(**Y**,**X**)}^*k*^. We do this for a number of *τ* values in the range (20 in our empirical studies) and select the optimal tuning parameter *τ* as:

6 $$ \tau_{\text{opt}} = \min_{\tau}\left\{\text{MSPE}(\tau)\right\}.  $$

**Rank (*r*) selection:** In our discussions so far, we have treated the rank (*r*) as known. In practice, it needs to be estimated from the data. There are many methods proposed to estimate *r* in the literature, see for instance [15] and [29], and the references therein. In our empirical studies, we again use cross-validation to estimate the rank. More specifically, we choose an estimate $\widehat {r}$ such that

$$\widehat{r} = \min_{r} \left\{ \text{MSPE}\left(\tau_{\text{opt}},r\right)\right\}, $$

where MSPE(*τ*_opt_,*r*) is the MSPE for a given *r* and the optimal tuning parameter as selected by (6). The value of *r* in practice is small, often times between 1 and 3. In our empirical simulations, we try values *r* in {1,⋯,10}. If the optimal value of *r* obtained by the cross-validation approach is close to 10, one could expand the range. Our empirical results (Table 1 in Additional file 1) show that this approach works well.

## Results

### Simulation studies

In this section, we assess the finite sample performance of the proposed coordinate-independent sparse estimation method for reduced-rank regression (CISRRR). We assess, and compare, estimation, prediction and variable selection performances. Estimation and prediction performances are evaluated using, respectively,

7 $$ \Delta = \left\| \mathbf{C} - \widehat{\mathbf{C}} \right\|_{\text{F}}^{2}/(pq) \quad \text{and} \quad \text{MSPE} = \left\|\mathbf{Y}_{\text{t}} - \widehat{\mathbf{Y}}_{\text{t}} \right\|_{\text{F}}^{2}/(n_{t}q)  $$

where $\widehat {\mathbf {Y}}_{\text {t}} = \mathbf {X}_{\text {t}}\widehat {\mathbf {C}}, n_{\text {t}}$ is the test set sample size, and ∥.∥_F_ represents the Frobenius norm. Variable selection performance is evaluated using true positive rate (TPR), the ratio of truly important variables that the method selects as important, and false positive rate (FPR), the ratio of unimportant predictors that the method selects as important. TPR values close to one and FPR values close to zero indicate a better variable selection performance. In all our simulation settings, to minimize the effect of parameter tuning, we generate a large test set (with 10,000 observations), a strategy which was also employed by [16] and [18]. For our method, the tuning parameter candidates are taken at a grid of 20 equally spaced values between (0, *τ*_max_], where *τ*_max_ is as defined in the tuning parameter selection section. Also, unless otherwise specified, the tuning parameter is chosen by 5-fold cross-validation as described in tuning parameter selection section. We repeat the simulation process 50 times and report results in the form of boxplots of the corresponding values.

We compare our method, i.e. Algorithm 1, with a number of state-of-the-art competing sparse estimation methods for multivariate linear regression. The first competing method we consider is the signal extraction approach for sparse multivariate response regression (SiER) by [20]. This method exploits the reduced rank structure by assuming there exist matrices **A** and **B** such that **C**=**BA**^⊤^, and seeks such **A** and **B** that lead to the best rank *r* approximation of the signal matrix **XC**. We use the SiER package in R to implement this method [20], using the “cv.SiER” function with 5-fold cross-validation to select the tuning parameters. The second competing method we consider is the regularized multivariate regression for identifying master predictors (remMap) by [30]. This method does not assume the reduced rank structure and solves a penalized least squares problem with both row-wise and element-wise sparsity imposed on the coefficient matrix. We use the remMap package in R to implement this method [30]. The third competing method is the subspace assisted regression with row-sparsity (SARRS) method by [18], which was extended to yield row and column sparse estimators in [19]. SARRS is carried out by Algorithm 1 in [18]. The fourth competing method is the sparse partial least squares (SPLS) method due to [31] which identifies sparse latent components by maximizing the covariance between them and the responses with sparsity inducing penalty imposed. We implement SPLS using the spls package in R. We use the function “cv.spls” with 5-fold cross-validation to select the tuning parameters, with the number of components *K* selected from {1,⋯,10} and the thresholding parameter *η* selected from {0.1,⋯,0.9}. We note again that, for a fair comparison, we use the tuning parameter selection methods presented in the respective papers.

We compare the methods under different model parameter settings as characterized by the covariance matrix of the predictors, as well as different rank values, and *signal-to-noise* ratios. We adopt simulation settings from [16], which were also adopted by [18]. The rows of the design matrix **X** are i.i.d. random vectors sampled from a multivariate Gaussian distribution with zero mean vector and covariance matrix **Σ**, with **Σ**_*ij*_=*ρ*^|*i*−*j*|^. The coefficient matrix $\mathbf {C} \in \mathbb {R}^{p \times q}$ has the form

$$\mathbf{C} = \left[\begin{array}{ll} \mathbf{C}_{1} \\ \, \, \mathbf{0} \end{array}\right] = \left[\begin{array}{ll} b \mathbf{B}_{0}\mathbf{B}_{1} \\ \quad \mathbf{0} \end{array}\right], $$

with $b > 0, \mathbf {B}_{0} \in \mathbb {R}^{s \times r}$ and $\mathbf {B}_{1} \in \mathbb {R}^{r \times q}$, where all entries in **B**_0_ and **B**_1_ are i.i.d. random numbers from *N*(0,1). Large value of *b* correspond to a large signal-to-noise ratio. We consider the following four cases.

1. *n*>*p*=*q*: *n*=100,*p*=25,*q*=25,*s*=15,*r*=5,*b*=0.2,0.4,*ρ*=0.1,0.5,0.9.
2. *q*<*n*<*p*: *n*=30,*p*=100,*q*=10,*s*=15,*r*=2,*b*=0.5,1,*ρ*=0.1,0.5,0.9.
3. *n*<*p*=*q*: *n*=30,*p*=100,*q*=100,*s*=15,*r*=2,*b*=0.5,1,*ρ*=0.1,0.5,0.9.
4. *n*<*p*<*q*: *n*=30,*p*=100,*q*=1000,*s*=15,*r*=5,*b*=0.5,1,*ρ*=0.1,0.5,0.9.

**Fig. 1:**
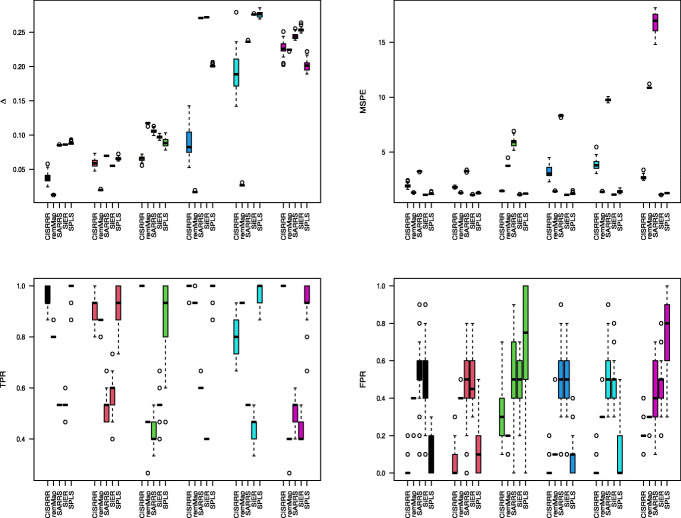
Simulation results for Gaussian errors under case 1. Reported results are 50 independent replications. *Δ* assesses estimation performance. MSPE is mean squared prediction error; TPR is true positive rate; FPR is false positive rate. Black for *ρ*=0.1,*b*=0.2; Red for *ρ*=0.1,*b*=0.4; Green for *ρ*=0.5,*b*=0.2; Blue for *ρ*=0.5,*b*=0.4; Cyan for *ρ*=0.9,*b*=0.2; Purple for *ρ*=0.9,*b*=0.4

**Fig. 2:**
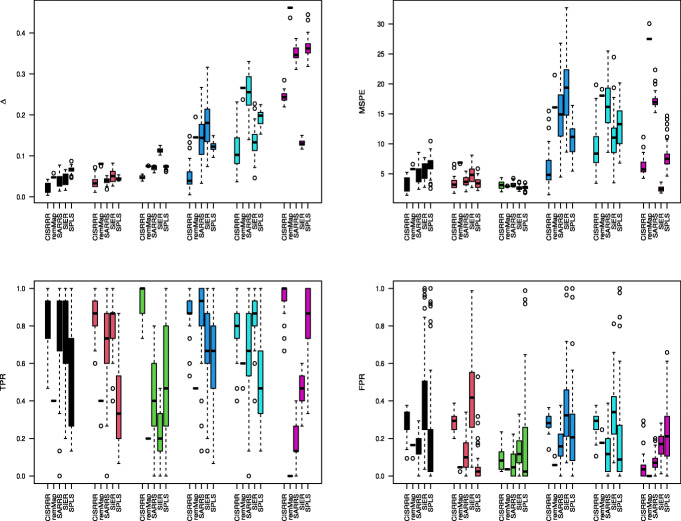
Simulation results for Gaussian errors under case 3. Reported results are 50 independent replications. *Δ* assesses estimation performance. MSPE is mean squared prediction error; TPR is true positive rate; FPR is false positive rate. Black for *ρ*=0.1,*b*=0.5; Red for *ρ*=0.1,*b*=1; Green for *ρ*=0.5,*b*=0.5; Blue for *ρ*=0.5,*b*=1; Cyan for *ρ*=0.9,*b*=0.5; Purple for *ρ*=0.9,*b*=1

We conduct additional simulations (refer to Figs. 1 and 2 in Additional file 1) where the error term matrix has entries from a non-Gaussian distribution. More specifically, we consider two additional noise distributions: $\mathbf {E}_{ij}\sim \sqrt {3/5}t_{5}$, and **E**_*ij*_∼3U[-1,1], where “3U[-1,1]” refers to the sum of three uniform [-1,1] random variables, and *t*_*ν*_ stands for a *t*-distribution with *ν* degrees of freedom.

### Simulation results

**Fig. 3:**
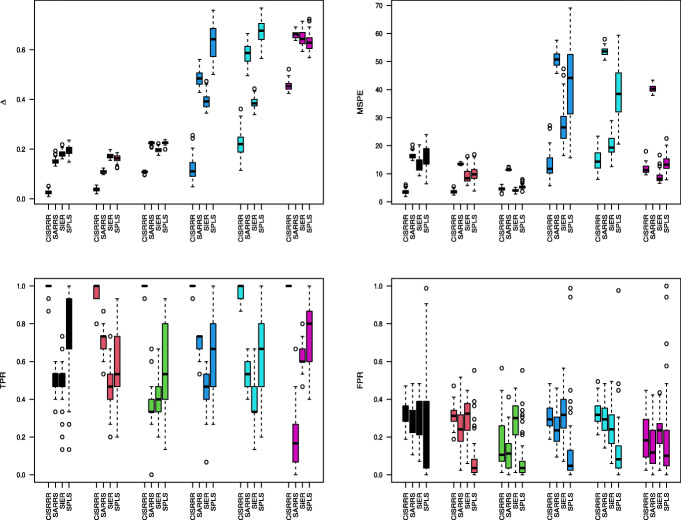
Simulation results for Gaussian errors under case 4. Reported results are 50 independent replications. *Δ* assesses estimation performance. MSPE is mean squared prediction error; TPR is true positive rate; FPR is false positive rate. Black for *ρ*=0.1,*b*=0.5; Red for *ρ*=0.1,*b*=1; Green for *ρ*=0.5,*b*=0.5; Blue for *ρ*=0.5,*b*=1; Cyan for *ρ*=0.9,*b*=0.5; Purple for *ρ*=0.9,*b*=1

Figures 1, 2 and 3 report simulation results for the scenario where the noise matrix $\mathbf {E} \in \mathbb {R}^{n \times q}$ has i.i.d. *N*(0,1) entries. Figure 1 reports the results for case 1 (*n*=100,*p*=*q*=25), from which, we make the following observations. In terms of estimation and prediction performances, remMap outperforms all the other methods, especially when *ρ*=0.1 and 0.5. This is not surprising, as remMap does not impose the low-rankness assumption and the sample size dominates both *p* and *q*. However, our method (CISRRR) yields comparable results with remMap, and even outperforms it when *ρ*=0.9 when remMap appears to struggle - again perhaps because it is marginal model. We see that all the methods also perform reasonably well and comparably *ρ*=0.1. In terms of variable selection, our method yields the best results in all settings. It yields TPR values that are significantly higher than the TPR values of the other existing methods, and comparable (often better) FPR values. In fact, we see that the TPR values of our method are consistently around 1. We see that both SARRS and SiER struggle in this case, especially in terms of TPR. Figure 2 reports the results for case 3 (*n*=30,*p*=100,*q*=100). Here, we see that CISRRR outperforms all the other methods in estimation, prediction, and TPR performances. Even though the performances in TPR appear comparable to that of SARRS and SiER, it is seen that our method yields more stable results - with less variability. With respect to FPR, our method is inferior to remMap and SPLS, and to a lesser degree SARRS, especially in the *ρ*=0.1 and *ρ*=0.5 cases. However, both remMap and SPLS yield very inferior TPR values, an indication that they both yield very sparse estimates. The performances for case 2 (not reported to save space) are similar to the performances for case 3. Figure 3 reports the results for case 4 (*n*=30,*p*=100,*q*=1000). For this case, remMap did not produce results, since *q* is large and it does not make the reduced-rank assumption. Here again, we see that our method outperforms all the other methods in estimation, prediction, and TPR, in all the settings. The advantage of our method is, in fact, more pronounced in this case as it yields superior results consistently. Especially when the correlation structure among the predictors is higher (*ρ*=0.9 vs *ρ*=0.5 vs. *ρ*=0.1), we see that the other methods performances deteriorate but our method continues to perform well. These observations are true for estimation, prediction and TPR. However, our method pays some price in terms of FPR as it does not outperform any of the other methods in terms of FPR. As we indicated earlier, SPLS yields more sparse results and thus have lower TPR and lower FPR values. Overall, our method is shown to yield competitive, and often times superior, results.

In all the simulation settings that we consider, the estimation and prediction performances of the methods are better when the correlation structure among the predictors is weaker (*ρ*=0.1 vs *ρ*=0.5). Nevertheless, we see that our method continues to perform well, relative to the other methods. Overall, our methods yields competitive (often superior) results in estimation, prediction and TPR. In terms of FPR, it yields comparable performance, sometimes inferior to the best performing method. Furthermore, we observe that remMap performs well for the large *n* setting, and struggles when *q* is large since it does not induce the reduced-rank structure, as well as when *ρ* is large since it is a marginal model. Finally, it appears that, like remMap, SPLS performs well for the large *n* setting (case 1), but it struggles in variable selection when either *p* or *q* is large (cases 2, 3 and 4).

### Real data analysis: the atherosclerosis disease study

**Study goals and design:** We apply the proposed method for simultaneous analysis of genetic (single nucleotide polymorphisms, SNPs) and metabolomics data. Data were obtained from the Emory University and Georgia Tech Predictive Health Institute (PHI) study. Our goal in this section is to identify relevant SNPs, and corresponding genes, that are simultaneously associated with metabolites, and which can be used to predict 10-year risk for atherosclerosis diseases (ASCVD). Specifically, we seek to use metabolites to guide selection of SNPs that may be associated with ASCVD, and to explore an indirect relationship between metabolites and ASCVD through the genetic variants.

**SNP and metabolomics quality control and filtering:** We obtained genetic and metabolomics data from the Emory PHI study. Several studies point to the association between biomarkers of inflammation, and the risk of CVD [32, 33]. As such, recent effort has focused on identifying biomarkers of inflammation and characterizing their effect on CVD [34]. We therefore focused on inflammation-related genes, and the SNPs within these genes. Specifically, we pulled all SNPs in our data that were in gene regions found in the inflammation pathway from NCBI dbSNP; there were 262,157 SNPs. Of note, these SNPs may or may not be associated with ASCVD. For SNP quality control, please refer to the flow chart given in Fig. 3 in the web supplementary material (Additional file 1). We assumed an additive genetic model in which the genotypes were coded to count the number of minor alleles so “0” for both homozygous major, “1” for heterozygotes (1 major, 1 minor), and “2” for risk-allele homozygotes (minor alleles). We treated the genetic data as continuous.

We obtained metabolomics data on 6,010 m/z features. We removed features having more than 50% zeros and coefficient of variation ≥20*%*. This resulted in 272 m/z features for the analyses. Because of the skewed distributions of most metabolomic levels, we log2 transformed each feature. We standardized each feature to have mean zero and unit variance.

**Application of the proposed and competing methods:** We had matching genetic and metabolomics data on 121 subjects. We applied our method to the metabolomics (**Y**_121×272_) and genetic (**X**_121×1988_) data to identify subset of SNPs that are simultaneously associated with metabolomics data, and which potentially can predict ASCVD risk. We obtained 50 bootstrap training and testing datasets by sampling the dataset with replacement. Out of bag samples (samples in the original data but not in the bootstrap training sets) were considered as bootstrap testing sets. For each bootstrap dataset, we estimated the rank *r* of the coefficient matrix for the multivariate regression model using 5-fold cross-validation as described in the tuning parameter selection section. The rank of the coefficient matrix was estimated to be $\widehat {r}=2$. Next, we apply the methods to the training data to obtain the estimated coefficient matrix, $\widehat {\mathbf {C}}$, which yields the predicted values for the test metabolite samples, $\widehat {\mathbf {Y}}_{\text {test}}$. We use **Y**_test_ and $\widehat {\mathbf {Y}}_{\text {test}}$ to compute the test MSPE, as given in (7). We record the number of non-zero rows of the estimated coefficient matrix (selected SNPs) for each method and for each bootstrap testing datasets. The averages are reported in Table 1.

**Table 1:** Average MSPEs and average number of selected SNPS (non-zero rows) for the competing methods from 50 independent bootstrap replications

	CISRRR	**SARRS**	**SIER**	**SPLS**	**remMap**
MSPE	1.013	1.038	1.035	1.032	****
# Selected SNPs	72.820	146.00	274.4	278.420	****
SARRS	8				
SiER	6	5			
SPLS	2	3	7		

The bottom half of the table presents the number of overlapping SNPs in the respective top 15 SNPs for the methods

Since our goal is to identify potential novel genetic variants that are linked with m/z features, and which could predict ASCVD risk, we considered the following analyses after identifying potential SNPs. The 10-year ASCVD risk score was dichotomized into low- vs moderate- to high-risk ASCVD. Specifically, ASCVD risk score ≥5.0*%* was considered moderate to high-risk, and ASCVD risk score <5*%* was considered low-risk [35, 36]. A weighted genetic-risk score (GRS) that utilizes the SNPs appearing at least 90% (>45 times) out of the 50 bootstrap datasets was calculated by multiplying the logarithm of the odds ratio for that particular SNP by 0, 1, or 2 depending on the number of risk alleles carried by each subject using the whole data. The log odds ratio from each bootstrap was estimated for each of the 15 SNPs, and a weighted mean of the estimates was used in calculating the genetic risk score. We further considered whether including the GRS to a model that used well-established risk factors (age and/or sex) improved predictive ability.

We explored causal association between metabolites and ASCVD risk, after adjusting for age and sex, by utilizing the GRS as an instrument in the causal pathway using Mendelian randomization [37]. Specifically, if the genetic risk score is statistically significantly associated with certain metabolites and is associated with ASCVD risk, then this would provide supportive evidence for a potential causal effect of that metabolite on ASCVD. In the Mendelian randomization analyses, we adopted the two-stage process outlined in [37]. In the first stage, we considered a linear regression model of each m/z features on the GRS controlled for age and sex, and we obtained fitted values for m/z features that showed significant association with the GRS (Benjamini-Hochberg False Discovery Rate [38] *p*-value <=.0004). In the second stage, these fitted values were included in a logistic regression model of ASCVD risk on the fitted values, and the effect on ASCVD risk was assessed after adjusting for age and sex.

### Results

**SNPs identified using the proposed and existing methods:** We investigate the SNPs identified by the proposed method, and the corresponding genes, with respect to their potential effect on ASCVD. Table 1 reports the average MSPEs and average model size (number of selected SNPs). Our proposed method identified 15 SNPs that were selected in at least 90% of the 50 independent replications (see web supplementary material). Of these selected 15 SNPS, 8 appear in the top 15 most selected SNPs by SARRS, 6 appear in the top 15 most selected SNPs by SiER, and 2 appear in the top 15 most selected SNPs by SPLS.

Table 2 in the web supplementary file shows the least squares means for the 15 SNPs identified by our method - the predicted population means for 10-year ASCVD risk, after adjusting for age and sex. For instance, the *rs1286264* SNP located on chromosome 14 is an intron variant found in the protein coding gene Ribosomal Protein S6 Kinase A5 (RPS6KA5). From our data, individuals with two risk alleles of this polymorphism are more likely to have lower adjusted 10-year ASCVD risk score least squares means compared with individuals with normal alleles or 1 risk and 1 normal alleles. A weighted genetic risk score developed using the 15 SNPs was significantly associated with ASCVD risk, after adjustment for age and sex (*p*-value <0.001) (Table 2 below). The predictive ability of the GRS + traditional risk factors was assessed with the area under the curve (AUC) from a receiver operating characteristics curve. We note from Table 2 that including the GRS improved AUC in both models. The difference between GRS + traditional risk factors model and only the traditional risk factor model were both statistically significant (*p*-value = 0.0134 for Model 1; *p*-value = 0.0104 for Model 2). Our findings suggest that a unit increase in the GRS increased the risk for ASCVD with an OR of 2.348 (95% CI: 1.599, 4.132) after controlling for age and sex. Intriguingly, when we dichotomize the GRS, with a high risk score >75th percentile, and low risk score ≤ 75th percentile, we find that the odds for ASCVD risk in the high genetic risk group was about 5 times the odds for ASCVD risk in the low genetic risk group, after controlling for age and sex (OR =5.076, *p*-value.0005, 95% CI: 1.77,14.49).

**Table 2:** Predicting ASCVD risk with GRS (genetic risk score) and traditional risk factors

	**Model 1**				**Model 2**	
	OR	*p*-value	CI	OR	*p*-value	CI
GRS	2.354	<.001	(1.466, 3.779)	2.348	<.001	(1.460, 3.776)
age	1.155	<.001	(1.065, 1.254)	1.158	<.001	(1.067, 1.256)
sex (M vs F)				0.771	0.6954	(0.242, 2.459)
AUC	**0.8428 vs 0.743 (Ref)**				**0.8441 vs 0.743 (Ref)**	

Ref represents reference ROC; model with no risk score

**Mendelian randomization exploratory analysis:** Here, we sought to tie the genetic risk score to m/z features and to explore causal association of m/z features to ASCVD risk. The genetic risk score served as an instrument to estimate the effect of the m/z features on ASCVD risk, after adjustment for age and sex. In this exploratory analysis, our findings suggested that the Dehydroalanine compound (C02218) is a possible risk factor for ASCVD. Specifically, the amino acid, Dehydroalanine, belonging to the Cysteine (Cys) and methionine (Met) metabolism pathway increased ASCVD risk with an odds ratio of 23.204 (95% CI: 4.106, 131.124) per SD increase in the log2 predicted plasma levels, after controlling for age and sex. Some research studies have documented the negative health consequences including elevated risk for cardiovascular diseases with high-intakes of both Met and Cys [39–41]. Our findings suggest an indirect association between Dehydroalanine amino acid and ASCVD risk through these genetic variants.

## Conclusion

We sought out to develop a method for identifying potential genetic variants that are associated with metabolites and have a predictive value beyond some established risk factors. We framed this as a two stage analysis: in the first stage we identified SNPs that were associated with the metabolites using dimension reduction techniques proposed in this article. In the second stage we used the selected SNPs as instruments to explore the cause-effect association of the selected metabolites with ASCVD. To handle the large number of SNPs and metabolites, we used sparse reduced-rank regression and proposed a new estimation method for the coefficient matrix using a group Dantzig type formulation. The proposed formulation is convex and can be solved using readily available solvers, such as the CVX toolbox in MATLAB. We carried out extensive simulation study to assess its finite sample performance, and compared it to other existing state-of-the-art methods.

In the second stage, we developed a genetic risk score comprised of 15 genetic variants and we assessed whether including the risk score in a model with well-established risk factors (age/sex) improved predictive ability. Our findings suggested a potential utility of the genetic risk score as it improved predictive ability. We used Mendelian randomization to explore association of a metabolite with ASCVD through the genetic risk score. Our analysis revealed a possible indirect association between the Dehydroalanine amino acid and ASCVD using the genetic risk score in the causal pathway, suggesting a potential role of Dehydroalanine on ASCVD risk through the genetic risk score. We note that our findings are just exploratory, as we lacked an independent data set to validate our results. Nevertheless, our results add to the literature on possible genetic variants that could be used in addition to established risk factors to improve prediction of atherosclerosis cardiovascular disease.

In our proposed method, we only focus on sparsity on **B**; this amounts to selection of predictors. One would be able to induce sparsity on **A** to select responses. We have not pursued this idea in this approach, but we believe it will be interesting to do so in the future.

## Supplementary information

**Additional file 1** Additional simulations and data analysis. We present additional simulations to assess the robustness of the proposed method when the error terms are non-Gaussian. In addition, in this file we report a flow-chart for the SNP selection process, and a table (Table 2) with the least squares means for the 15 SNPs identified by our method - the predicted population means for 10-year ASCVD risk, after adjusting for age and sex.

**Additional file 2** Software. Matlab codes that implements the proposed CISERRR algorithm.

## Data Availability

Matlab codes for implementing the method are available as Additional File 2. The gene expression, metabolomics, and clinical data were provided to us by the Emory Predictive Health Institute, and therefore cannot be made publicly available. As part of their data agreement consent, “Emory University and the Predictive Health Initiative retain ownership rights to all provided data regardless of the purpose or outcome of any subsequent publications or collateral works”. (http://predictivehealth.emory.edu/documents/CHDWB_EmoryUniversity_DataUseRequestForm.pdf) The data may be requested from https://redcap.emory.edu/surveys/?s=7PYMFLHYTL.

## Abbreviations

ASCVD: Atherosclerosis cardiovascular diseases
SNPs: Single nucleotide polymorphisms
AUC: Area under receiver operating characteristic curves
GRS: Genetic risk score
MSPE: Mean squared prediction error
TPR: True positive rate
FPR: False positive rate
