# Supplementary material for: Sparse reduced-rank regression for integrating omics data
Source: BMC Bioinformatics. 2020 Jul 3;21:283. doi: 10.1186/s12859-020-03606-2 (PMC7333421; doi:10.1186/s12859-020-03606-2)
Supplement: Supplementary file 1 — Additional file 1 Additional simulations and data analysis. We present additional simulations to assess the robustness of the proposed method when the error terms are non-Gaussian. In addition, in this file we report a flow-chart for the SNP selection process, and a table (Table 2) with the least squares means for the 15 SNPs identified by our method - the predicted population means for 10-year ASCVD risk, after adjusting for age and sex. [file 12859_2020_3606_MOESM1_ESM.pdf]

Supplementary Materials for: Sparse reduced-rank regression for integrating omics data

Haileab Hilafu *   Sandra Safo †   and   Lillian Haine ‡

May 15, 2020

Additional Simulations with Non-Gaussian Error Terms

Here we present additional simulations to assess the robustness of the proposed method when the error terms follow a non-Gaussian distribution. To this end, we consider two different noise distribution settings: $\mathbf{E}_{ij} \sim \sqrt{3/5}t_5$ (Figure 1), and $\mathbf{E}_{ij} \sim$ 3U[-1,1] (Figure 2), where "3U[-1,1]" refers to the sum of three uniform [-1,1] random variables, and t_ν stands for a t-distribution with ν degrees of freedom.

Overall, these results show similar patterns as in the Gaussian errors (Figure 1 in the paper). Our proposed method continues to perform competitively, and performs atleast as well as the best competing method in terms of TPR. In addition, the performances of our proposed method for these non-Gaussian error distribution cases are very similar to its performances, in all model settings, indicating that the method is robust to the normal error distribution assumption we utilize for the theoretical analysis.

*Assistant Professor, Department of Business Analytics and Statistics, University of Tennessee, Knoxville, TN 37996. E-mail: hhilafu@utk.edu

†Assistant Professor, Division of Biostatistics, University of Minnesota, 420 Delaware St. SE, Minneapolis, MN 55123. E-mail: ssafo@umn.edu

‡Graduate Student, Division of Biostatistics, University of Minnesota, 420 Delaware St. SE, Minneapolis, MN 55123. E-mail: haine108@umn.edu

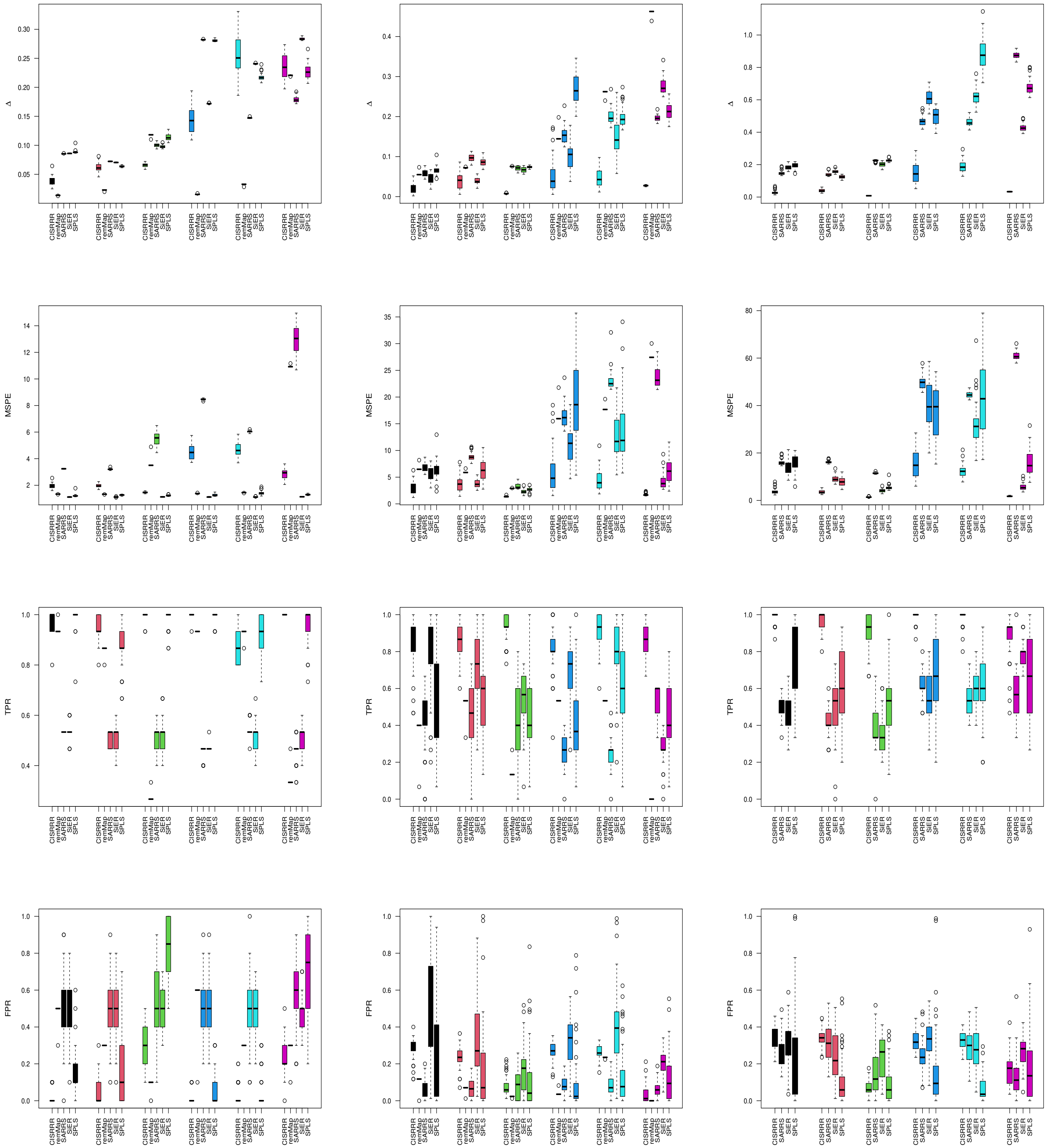

Figure 1: Simulation results for $\mathbf{E}_{ij} \sim \sqrt{3/5}t_5$. First column results are for case 1 ($n = 100; p = q = 25; r = 5$), second column results are for case 3 ($n = 30; p = 100; q = 100; r = 2$), and third column results are for case 4 ($n = 30; p = 100; q = 1000; r = 5$). Reported results are from 50 independent replications. Black for $\rho = 0.1, b = 0.2$; Red for $\rho = 0.1, b = 0.4$; Green for $\rho = 0.5, b = 0.2$; Blue for $\rho = 0.5, b = 0.4$; Cyan for $\rho = 0.9, b = 0.2$; Purple for $\rho = 0.9, b = 0.4$.

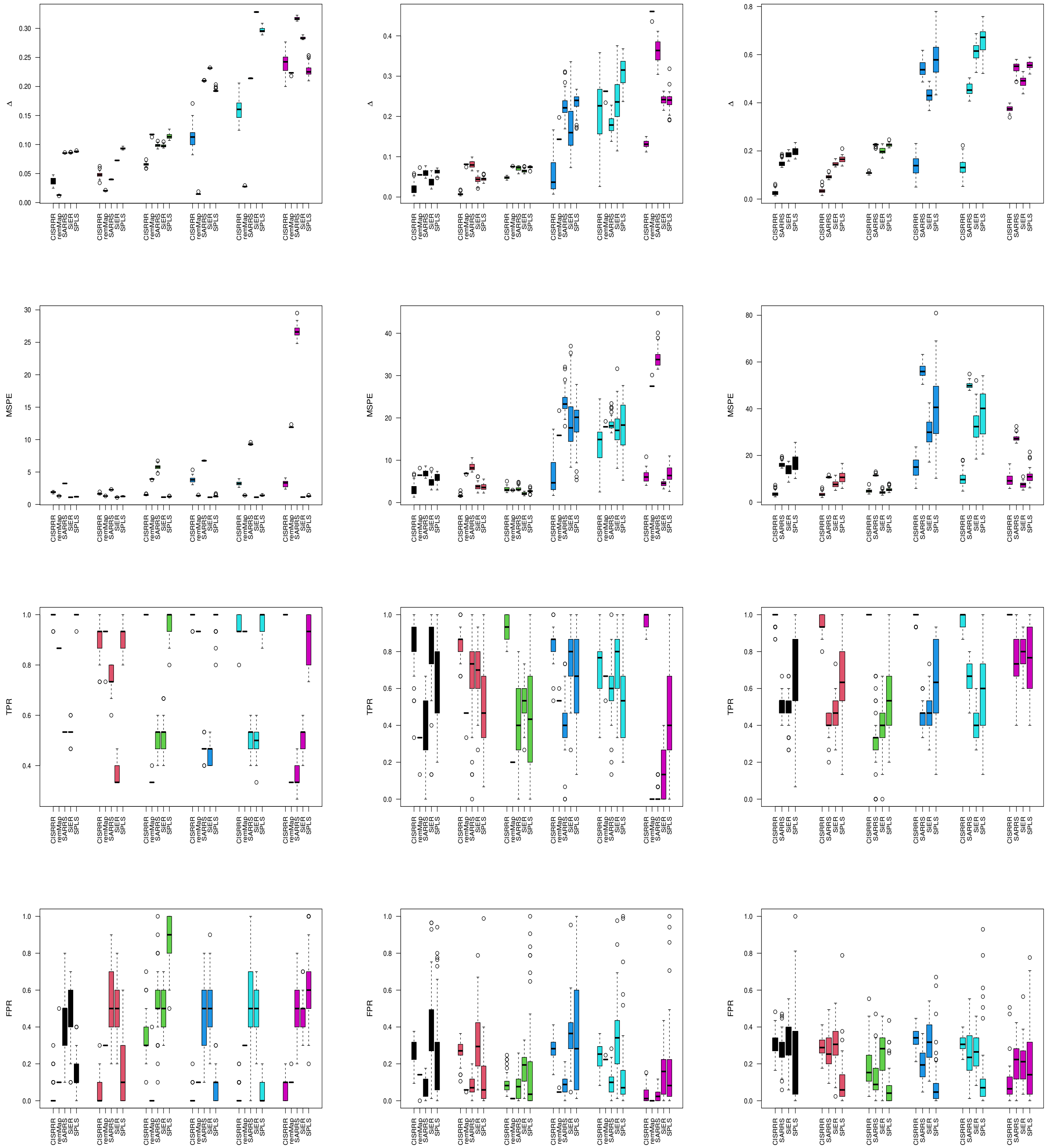

Figure 2: Simulation results for $\mathbf{E}_{ij} \sim 3U[-1,1]$. First column results are for case 1 ($n = 100; p = q = 25; r = 5$), second column results are for case 3 ($n = 30; p = 100; q = 100; r = 2$), and third column results are for case 4 ($n = 30; p = 100; q = 1000; r = 5$). Reported results are from 50 independent replications. Black for $\rho = 0.1, b = 0.2$; Red for $\rho = 0.1, b = 0.4$; Green for $\rho = 0.5, b = 0.2$; Blue for $\rho = 0.5, b = 0.4$; Cyan for $\rho = 0.9, b = 0.2$; Purple for $\rho = 0.9, b = 0.4$.

Simulations To Assess Rank Selection

Here, we conduct simulation studies to assess the performance of the cross-validation approach to estimate the rank, described in the Method Section. Table 1 below presents the results, where the error terms are drawn from the normal distribution. The reported values are `mean±standard deviation` over 50 independent replications. In general, we see that the approach performs reasonably well in estimating the rank. Interestingly, we observe that the approach appears to slightly over estimate the rank in all the reported setting.

Table 1: `Mean±Standard deviation` of the estimated rank via the cross-validation approach in Section , from 20 independent replications.

		$\mathbf{E}_{ij} \sim N(0,1)$	$\mathbf{E}_{ij} \sim t_3$	$\mathbf{E}_{ij} \sim 3U$
$(n = 100, p = 25, q = 25, b = 0.4, \rho = 0.5)$	$r = 5$	5.55±1.23	5.65±1.27	5.70±1.26
$(n = 30, p = 100, q = 10, b = 1, \rho = 0.5)$	$r = 2$	2.60±0.82	2.25±0.72	2.60±0.75
$(n = 30, p = 100, q = 100, b = 1, \rho = 0.5)$	$r = 2$	2.85±0.93	2.60±0.88	3.35±1.13
$(n = 30, p = 100, q = 1000, b = 1, \rho = 0.5)$	$r = 5$	5.65±0.93	5.95±1.09	5.50±0.76

1 Real Data Analysis

Table 2 shows the least squares means for three SNPs (complete table in web supplementary material) - the predicted population means for 10-year ASCVD risk, after adjusting for age and sex. The *rs4925112* SNP located on chromosome 17 is a coding synonymous polymorphism that occurs when there is a change from the "C" to the "T" nucleotide substitution in the sequence. Table 2 shows the least squares means - the predicted population means for 10-year ASCVD risk, after adjusting for age and sex. From our data, individuals with the "TT" genotype (risk allele) are more likely to have higher adjusted 10-year ASCVD risk score least squares means compared with individuals with "CT" or "CC" genotypes (see Table 2).

Figure 3: Steps used in pre-processing SNPs for analysis.

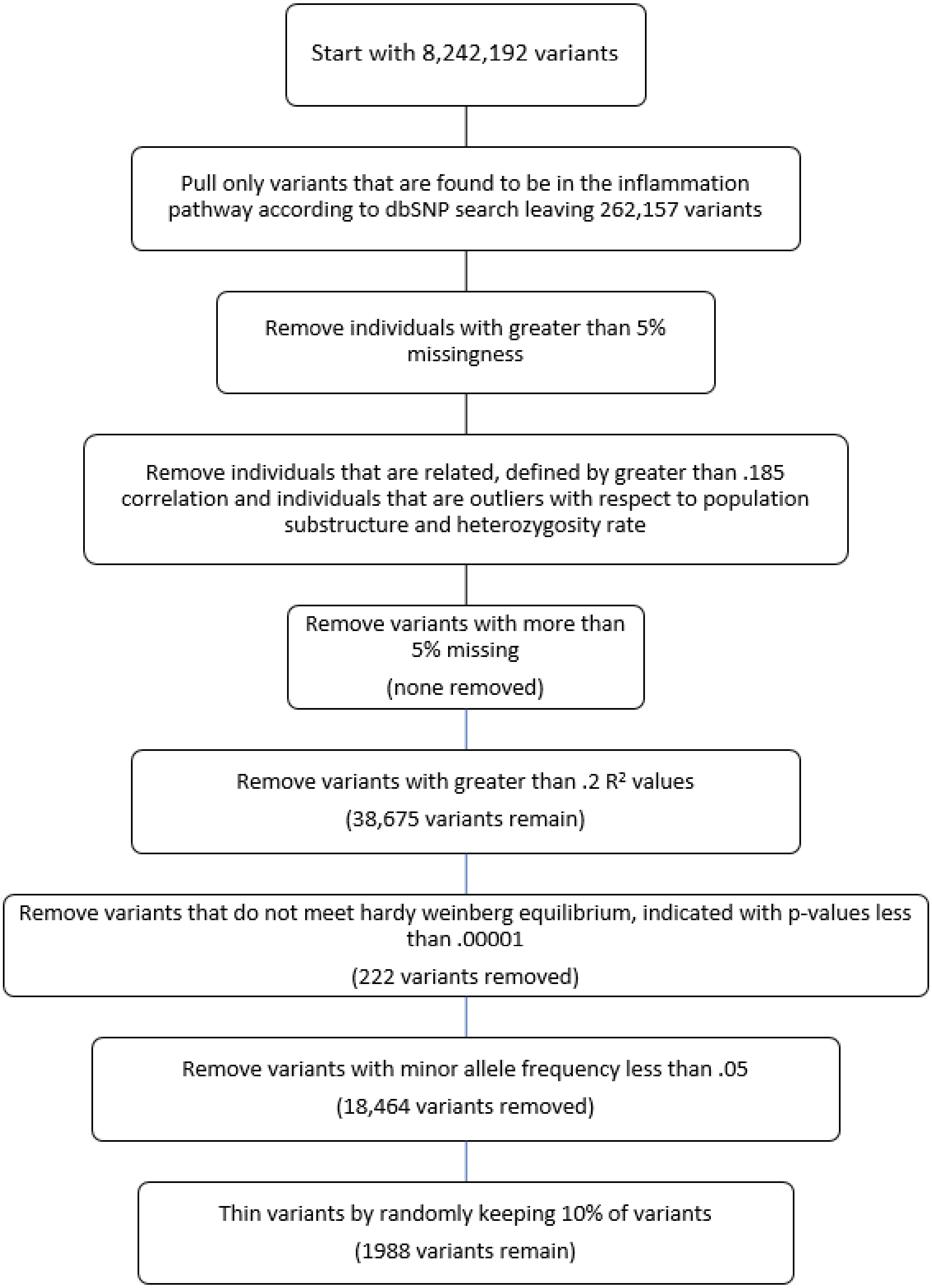

Table 2: Adjusted (age and sex) 10-year ASCVD Risk Score Least Squares Means for SNPs showing significant association with ASCVD risk using multivariate linear regression. ∗ indicates minor allele frequency; + indicates adjusted least squares means for genotype in the order of major allele homozygote (normal allele), heterozygote and, minor allele homozygote (risk allele).

SNP	MAF*	Chromosome	Nearby Gene	Adjusted LS Means+	LCI	UCI
rs651646	0.459	11	FOLR2	2.964	1.661	4.267
				3.486	2.400	4.571
				3.687	2.329	5.045
rs114686998	0.491	2	CPS1	3.574	2.213	4.936
				3.664	2.675	4.654
				2.484	0.955	4.014
rs4721	0.433	7	RARRES2	3.439	2.177	4.701
				3.630	2.619	4.642
				2.659	1.051	4.268
rs644818	0.498	11	FLI1	3.249	1.878	4.620
				3.855	2.797	4.913
				2.777	1.451	4.103
chr13.36402817.I	0.296	13	DCLK1	3.915	2.926	4.903
				2.810	1.635	3.985
				2.855	0.873	4.836
rs9634642	0.479	13	ABCC4	2.526	1.057	3.994
				3.699	2.691	4.707
				3.575	2.225	4.926
rs8054681	0.393	16	ZFPM1	3.246	2.088	4.404
				3.786	2.739	4.833
				2.608	0.915	4.300
rs1286264	0.409	14	RPS6KA5	3.797	2.565	5.029
				3.495	2.483	4.507
				2.359	0.666	4.052
rs1053900	0.497	14	MEG3	2.583	1.375	3.792
				3.928	2.763	5.092
				3.685	2.368	5.003
rs6083801	0.465	20	ABHD12	2.943	1.654	4.231
				3.896	2.834	4.958
				3.001	1.538	4.464
rs1033583	0.313	6	DLL1	3.220	2.144	4.295
				3.689	2.634	4.744
				2.773	0.556	4.991
rs2702945	0.302	8	DEFB1	3.300	2.296	4.304
				3.457	2.346	4.567
				3.636	1.047	6.226
rs11782190	0.499	8	PTK2B	2.799	1.474	4.124
				3.756	2.781	4.732
				3.226	1.610	4.841
rs10843050	0.248	12	PZP	3.725	2.721	4.730
				3.117	1.997	4.237
				2.209	-0.728	5.146
rs216762	0.481	21	APP	3.926	2.589	5.263
				3.372	2.407	4.337
				2.630	0.959	4.300
